# Combined *de novo* transcriptomic and physiological analyses reveal RyALS3-mediated aluminum tolerance in *Rhododendron yunnanense* Franch

**DOI:** 10.3389/fpls.2022.951003

**Published:** 2022-08-10

**Authors:** Yan-Xia Xu, Yun-Sheng Lei, Shan-Xia Huang, Jing Zhang, Zi-Yun Wan, Xiang-Tao Zhu, Song-Heng Jin

**Affiliations:** ^1^Jiyang College, Zhejiang A&F University, Zhuji, China; ^2^Zhejiang Provincial Key Laboratory of Forest Aromatic Plants-based Healthcare Functions, Zhejiang A&F University, Lin’an, China; ^3^The Nurturing Station for the State Key Laboratory of Subtropical Silviculture, School of Forestry and Biotechnology, Zhejiang A&F University, Lin’an, China

**Keywords:** acid soil, aluminum toxicity, exclusion, immobilization, organic acids, *Rhododendron yunnanense* Franch, transporter protein

## Abstract

*Rhododendron* (Ericaceae) not only has ornamental value, but also has great medicinal and edible values. Many *Rhododendron* species are native to acid soils where aluminum (Al) toxicity limits plant productivity and species distribution. However, it remains unknown how *Rhododendron* adapts to acid soils. Here, we investigated the physiological and molecular mechanisms of Al tolerance in *Rhododendron yunnanense* Franch. We found that the shoots of *R. yunnanense* Franch did not accumulate Al after exposure of seedlings to 50 μM Al for 7 days but predominantly accumulated in roots, suggesting that root Al immobilization contributes to its high Al tolerance. Whole-genome *de novo* transcriptome analysis was carried out for *R. yunnanense* Franch root apex in response to 6 h of 50 μM Al stress. A total of 443,639 unigenes were identified, among which 1,354 and 3,413 were up- and down-regulated, respectively, by 6 h of 50 μM Al treatment. Both Gene Ontology (GO) enrichment and the Kyoto Encyclopedia of Genes and Genomes (KEGG) pathway enrichment analyses revealed that genes involved in “ribosome” and “cytoskeleton” are overrepresented. Additionally, we identified Al-tolerance homologous genes including a tonoplast-localized ABC transporter RyALS3; 1. Overexpression of RyALS3; 1 in tobacco plants confers transgenic plants higher Al tolerance. However, root Al content was not different between wild-type plants and transgenic plants, suggesting that RyALS3; 1 is responsible for Al compartmentalization within vacuoles. Taken together, integrative transcriptome, physiological, and molecular analyses revealed that high Al tolerance in *R. yunnanense* Franch is associated with ALS3; 1-mediated Al immobilization in roots.

## Introduction

It has been estimated that nearly 30% of the world’s ice-free lands and 50% of potential arable lands belong to acid soils (von Uexküll and Mutert, 1995). At global scale, these acid soils are mainly distributed in two belts, i.e., the northern cool temperate zone and the southern tropic and subtropic zones (von Uexküll and Mutert, 1995). Due to the reduced pH of acid soils, aluminum (Al) in aluminosilicates or oxides is exchanged by proton and released into soil solution in ionic forms, which has been recognized as an important factor limiting plant productivity and species distribution (Kochian, 1995).

To deal with Al stress, plants have developed Al resistance mechanisms that can be grouped into three strategies. The first is external exclusion based mainly on the exudation of substances that chelate Al to form non-toxic complexes (Yang et al., 2019). The second is internal tolerance relying on chelation and subsequent sequestration of Al within vacuoles (Ma et al., 2021). Finally, there are several cases describing the facilitated transportation of Al from root to shoot, thereby increasing Al resistance (Kochian et al., 2015). To date, a lot of genes have been functionally characterized in terms of Al tolerance in model plants and crops. For instance, genes responsible for citrate and malate efflux activated by Al have been reported belonging to multidrug and toxic compound extrusion (MATE) and aluminum-activated malate transporter (ALMT) protein family, respectively. In rice, plasma membrane-localized Nrat1, a member of natural resistance-associated macrophage protein (Nramp) family, and tonoplast-localized OsALS1, a half-size ATP-binding cassette (ABC) transporter, have also been characterized to be responsible for Al uptake across the plasma membrane and tonoplast, respectively (Xia et al., 2010; Huang et al., 2012). However, genes homologous to rice Nrat1 have not been present in Arabidopsis genome and an aquaporin AtNIP1;2 has been reported functioning as a bidirectional transporter for Al-malate complex (Wang et al., 2017; Chen et al., 2022). Recently, an ABC transporter protein, ALS3, has been reported to be localized at tonoplast and may function as an Al transporter in Arabidopsis (Godon et al., 2019). In addition to genes encoding transporter protein, a transcription factor (TF) SENSITIVE TO PROTEON RHIZOTOXICITY1 (STOP1) has been demonstrated as a master TF that regulates the expression of many downstream Al-tolerance genes (Sawaki et al., 2009). However, most research advances were achieved by studying crop plants such as wheat and rice and model plants such as Arabidopsis and tomato (Kochian et al., 2015; Yang et al., 2019; Jin et al., 2021, 2022; Chen et al., 2022). Al toxicity also occurs in forests because nearly 67% of acid soils are covered by forests and woodland (Brunner and Sperisen, 2013). Intriguingly, many woody plants grow vigorously in acid soils with high Al concentrations. Therefore, woody plants must have evolved sophisticated mechanisms to survive Al stress, some of which may be shared with those in crops, while others may be distinct.

The genus *Rhododendron* (Ericaceae) encompasses over 1,000 species and China is home to 571 species. Located in southwest China, Yunnan province, where acid soil predominates, is rich in genus *Rhododendron* (Geng, 2014). *Rhododendron* species in Yunnan province typically grows in mountainous sparse thicket or pine forest, preferring acid soils. Therefore, it has been frequently regarded as an indicator plant of acid soils. However, it remains unclear how they adapt to an Al toxic environment. In this present study, *R. yunnanense* Franch was investigated in terms of Al tolerance and physiological and molecular bases of Al tolerance. We reveal that a tonoplast-localized ALS3 protein confers high Al tolerance to *R. yunnanense* Franch, possibly *via* Al immobilization in roots.

## Materials and methods

### Plant materials and growth conditions

Seeds of *R. yunnanense* Franch were collected from Shizong County, Qujing City, Yunnan Province, China, in November, 2020. Seeds were fully imbibed with water and germinated in the mixture of peat soil and vermiculite (3, 1 w/w). After germination, the seedlings were transplanted to a pot containing 8 L of 1/5 Hoagland nutrient solution (pH 5.0) comprised 1.0 mM of Ca (NO_3_)_2_, 1.0 mM of KNO_3_, 0.4 mM of MgSO_4,_ 0.2 mM of (NH_4_)H_2_PO_4_, 20 μM of NaFeEDTA, 0.5 μM of MnCl_2_, 3.0 μM of H_3_BO_3_, 0.4 μM of ZnSO_4,_ 1 μM of (NH_4_)_6_Mo_7_O_24_, and 0.2 μM of CuSO_4_. After 2 months, the uniform seedlings were selected and subjected to Al treatment. The treatment solution was 1/5 Hoagland nutrient solution (pH 5.0), but (NH_4_)H_2_PO_4_ concentration was reduced to 10 μM. Al in the form of AlCl_3_ (50 mM in water) was added directly into the nutrient solution to reach the final concentrations. All experiments were carried out in an environment-controlled room with 16 h photoperiod and 24°C at daytime and 22°C at night.

### RNA isolation and transcriptome sequencing

After treatment, root apices were excised and frozen in liquid nitrogen for subsequent total RNA extraction by using an RNeasy Mini Kit (Tiangen). The extracted RNA was digested by RNase-free DNAase I (Qiagen) to remove residual DNA. For the cDNA library construction, mRNA was enriched and broken into short fragments in length, which was used for first-strand cDNA synthesis. Next, the second-strand cDNA was synthesized by using DNA Polymerase I, and the adaptor sequences were ligated to the cDNA fragments and PCR amplified to create the cDNA libraries. After library quality assessment, RNA-sequencing was performed on an Illumina Hiseq 2000 platform. The clean reads were deposited in the National Center for Biotechnology Information BioProject database.

### Transcriptome data processing and analysis

Clean reads were obtained after processing raw reads with in-house perl scripts. The DESeq R package (1.10.1) was employed for differential expression analysis between-Al and + Al libraries with an adjusted *p*-value <0.05. Gene Ontology (GO) enrichment analysis of the DEGs was carried out with the topGO R packages according to Kolmogorov–Smirnov test. The KEGG (Okuda et al., 2008) database^1^ was used for metabolic pathway enrichment analysis and KOBAS software (Xie et al., 2011) was adopted to analyze KEGG pathways enrichment for DEGs.

### qRT-PCR analysis

For qRT-PCR analysis, 2-month-old seedlings were subjected to 1/5 Hoagland nutrient solution (pH 5.0) with 10 μM (NH_4_)H_2_PO_4_ containing different Al concentrations for 6 h, or for different treatment times by exposing to 50 μM Al. After treatment, the root apex (1 cm) was excised for RNA extraction. First-strand cDNA was synthesized by PrimeScriptTM RT Master Mix (TaKaRa). qRT-PCR was performed on Roche LightCycle 480 II system (LC480, Basel, Switzerland) with SYBR Premix ExTaq kit (Takara). The PCR reaction condition was 95°C for 5 min, with 40 cycles of 95°C for 10 s, 58°C for 30 s, and 70°C for 10 s. ACTIN was used as a normalization control. All primers are listed in Supplementary Table S1. The 2^−ΔΔCt^ method was used to calculate relative gene expression. For each gene and sample, three biological and three technical replicates were analyzed.

### Subcellular localization analysis

The 459-bp coding DNA sequence of RyALS3;1 without the stop codon was PCR amplified using root cDNA as a template. Then, the PCR products were ligated into the binary vector (pCAMBIA1300) to produce the Pro35S::RyALS3;1::GFP construct, which was next transformed into Agrobacterium tumefaciens strain GV3101, followed by infecting 4-week-old tobacco leaves. For co-localization, the tonoplast marker (vac-rk CD3–975) was co-transformed with Pro35S::RyALS3;1::GFP construct (Nelson et al., 2007; Xu et al., 2019). After 48–72 h of infiltration, GFP and RFP signals were observed using confocal laser scanning microscopy (Zeiss Leica TCS SP5; Mannheim, Germany).

### Al tolerance in tobacco and Al content measurement

For constructing RyALS3;1 overexpression tobacco lines, the coding region of RyALS3;1 was PCR amplified by using gene-specific primer pairs (Supplementary Table S1) and cloned into pCAMBIA1300 vector after CaMV 35S promoter. The resultant plasmid was transformed into Agrobacterium tumefaciens strain GV1301, which was used to transform tobacco (*Nicotiana tabacum*) plants. Three transgenic tobacco (*Nicotiana tabacum*) lines overexpressing *R. yunnanense ALS3;1* and the wild type were used for Al-tolerance assessment. Evaluation of Al tolerance followed (Xia et al., 2010). In brief, when the primary root length reached 1 cm, the seedlings were transferred to the 1/5 Hoagland nutrient solution (10 μM NH_4_H_2_PO_4_) containing 0 or 5 μM Al (pH 5.0) for 4 days. After treatment, the root tip (0–1 cm) was excised, washed with distilled water, and dried. Then, the samples were digested by a mixture of 1:1 (v/v) H_2_SO_4_ and HNO_3_, and Al was quantified by an inductively coupled plasma atomic emission spectrometer (Thermo Jarrell Ash, Franklin, MA, USA).

## Results

### *Rhododendron yunnanense* Franch is highly tolerant to Al

**Figure 1:**
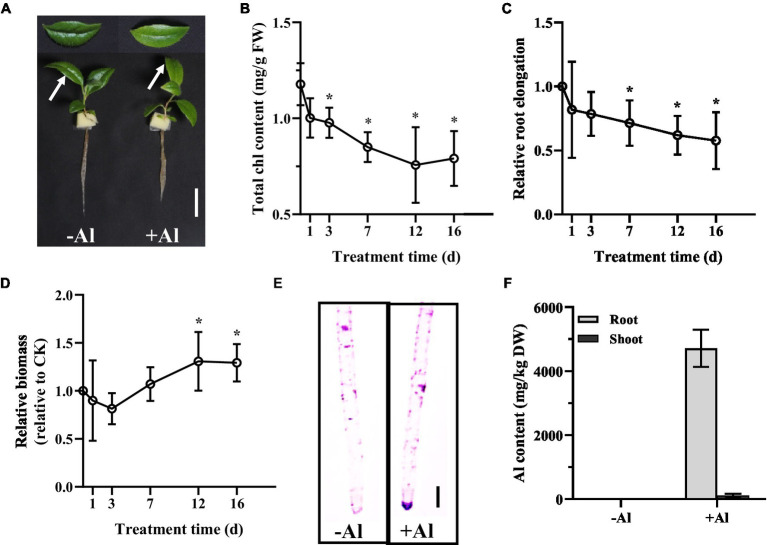
High Al tolerance in *R. yunnanense* Franch. Two-month-old seedlings were subjected to 1/5 nutrient Hoagland solution with or without 50 μM Al for 16 days. **(A)** Photo shows phenotype of seedlings without (−Al) or with (+Al) after 16 days. Bar = 1 cm. **(B)** Change of Chl content of newly expanded leaves during Al stress. **(C)** Relative root elongation. **(D)** Relative root biomass. **(E)** Hematoxylin staining of root apex after 7 days of Al treatment. **(F)** Al content in roots and shoots after 7 days of treatment. Asterisks (*) represent a significant difference in comparison with 0 μM Al control.

To examine the Al tolerance of *R. yunnanense* Franch, 2-month-old seedlings were transferred to1/5 strength Hoagland nutrient solution containing 0 or 50 μM Al (pH 5.0) over 16 days. Phosphate (Pi) concentration in the nutrient solution was reduced to 10 μM to avoid precipitation of Al by Pi. After 16 days of treatment, we observed leaf chlorosis and reduced primary root elongation for the seedlings (Figure 1A). Measurement of total chlorophyll (chl) content showed that a significant decrease of chl content appeared on day 3 and tended to decrease further over time (Figure 1B). However, the longest root was not significantly inhibited until day 7, and about a 40% reduction in the longest root elongation was observed on day 16 (Figure 1C). By contrast, the root biomass was increased by Al stress after 12 days of treatment (Figure 1D), suggesting that *R. yunnanense* Franch is highly tolerant to Al stress. Hematoxylin staining indicative of Al accumulation showed no significant binding of Al onto the root surface of *R. yunnanense* Franch (Figure 1E). It has been well-documented that external exclusion mechanisms play important roles in Al resistance (Yang et al., 2019). Thus, the hematoxylin staining that showed Al was not accumulated in the roots surface prompted us to further investigate whether Al exclusion was responsible for the high Al tolerance of *R. yunnanense* Franch. To this end, we determined Al content in both roots and shoots after 7 days of treatment. Interestingly, we found that the roots of *R. yunnanense* Franch accumulated as high as 5,000 mg kg^−1^ dry weight Al (Figure 1F), suggesting that it is not an external exclusion mechanism that contributes to high Al tolerance of *R. yunnanense* Franch. Moreover, Al did not accumulate in their leaves (Figure 1F), indicating that the effective translocation of Al from root to shoot is also not involved in its high Al tolerance. These observations indicate that *R. yunnanense* Franch is highly tolerant to Al and internal tolerance mechanisms potentially contribute to its high Al tolerance.

### Transcripts assembly and unigenes annotation

To discover the underlying molecular mechanisms of high Al tolerance of *R. yunnanense* Franch, transcriptome analysis was performed for the root apex of *R. yunnanense* Franch subjected to 0 or 50 μM Al for 6 h. After quality control of the raw RNA-seq data, approximately 103.8 million and 93.2 million clean reads with a mean length of 298 bp were obtained from the control and Al-treated libraries, respectively (Supplementary Table S2). Although chromosome-level genome assembly of *Rhododendron simsii* and *Rhododendron griersonianum* has been reported (Yang et al., 2020; Ma et al., 2021), the obtained reads of *R. yunnanense* Franch were poorly assembled on to these two genomes. Therefore, Trinity software was employed for the *de novo* assembly of transcripts in this study (Grabherr et al., 2011), which resulted in the production of a total of 645,145 transcripts with a mean length of 831 bp for *R. yunnanense* Franch roots (Supplementary Table S2). These transcripts represented 443,639 unigenes with a mean length of 647 bp (Supplementary Table S2). To annotate unigenes, all obtained unigenes sequences of *R. yunnanense* Franch were searched against protein databases. The results showed that 74,262, 176,416, 127,673, 120,822, 176,881, 100,616, 199,052, 142,259, and 215,407 unigenes were annotated by the COG database, GO database, KEGG database, KOG database, Pfam database, Swiss-Prot database, TrEBML database, eggNOG database, and Nr database, respectively. A total of 247,086 unigenes were annotated by at least one database (Supplementary Table S3). We checked the correlation coefficients among biological replicates; the high coefficient value indicated that the reproducibility is acceptable for the RNA-sequencing data (Supplementary Figure S1).

### Genome-wide gene expression of *Rhododendron yunnanense* Franch roots under Al stress

We identified differentially expressed unigenes (DEGs) based on the criteria of Log_2_ fold change (FC) ≥ 1 for upregulated genes or ≤−1 for downregulated genes and with adjusted *p*-value (*Q* value) < 0.05 by DEseq2 (Love et al., 2014). A total of 1,354 and 3,413 unigenes were found to be upregulated and downregulated, respectively, after 6 h of 50 μM Al treatment (Supplementary Figure S2A). Hierarchical cluster analysis of DEGs also showed that downregulated genes are much more common than upregulated genes in *R. yunnanense* Franch roots (Supplementary Figure S2B). The lists of both upregulated and downregulated unigenes are shown in Supplementary Tables S4, S5.

**Figure 2:**
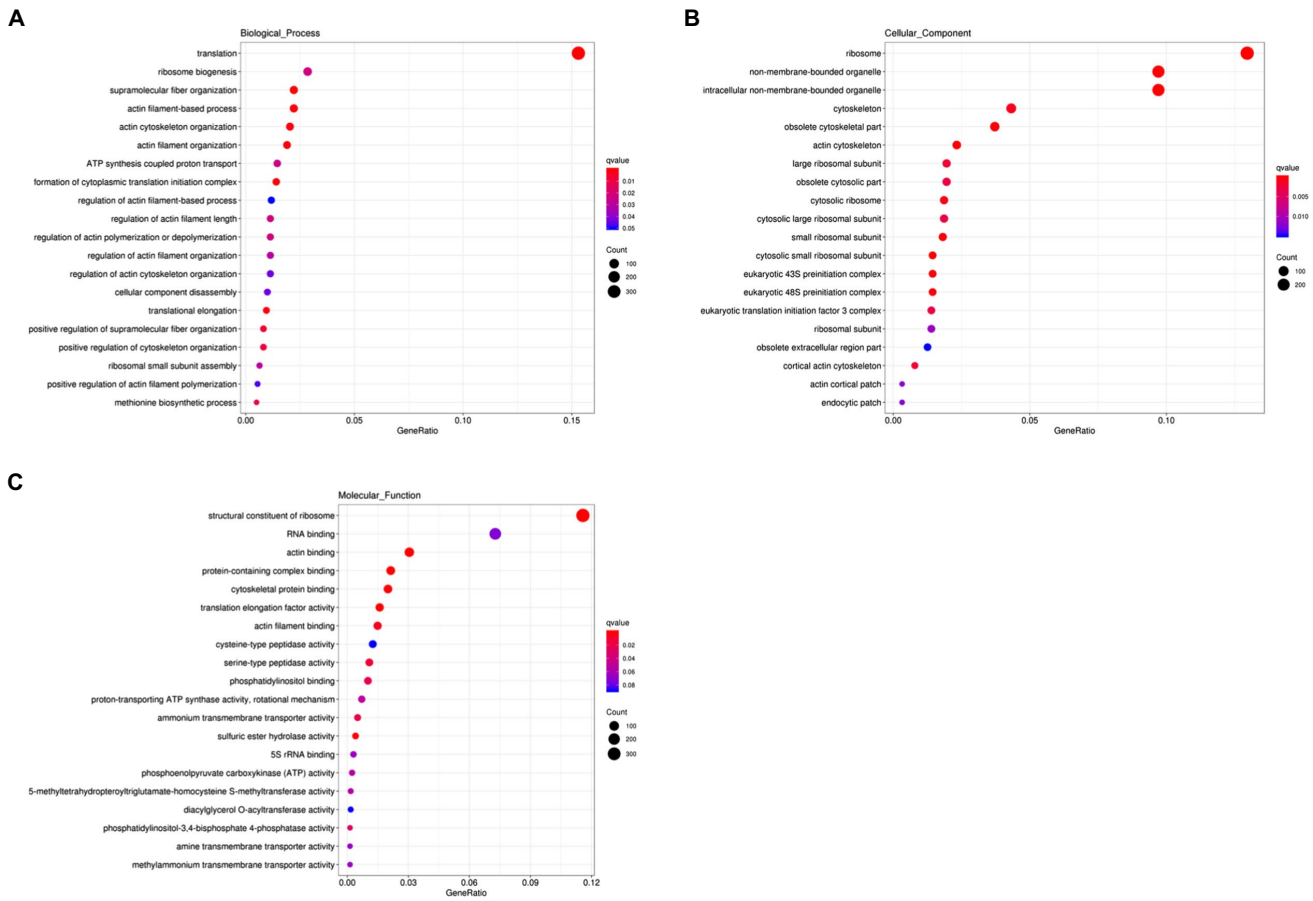
GO enrichment analysis of DEGs. **(A)** Biological process. **(B)** Cellular component. **(C)** Molecular function. The *y*-axis represents the process name. The *x*-axis is GeneRatio, that is, the proportion of the genes of interest annotated in this item to the number of all differentially expressed genes.

**Figure 3:**
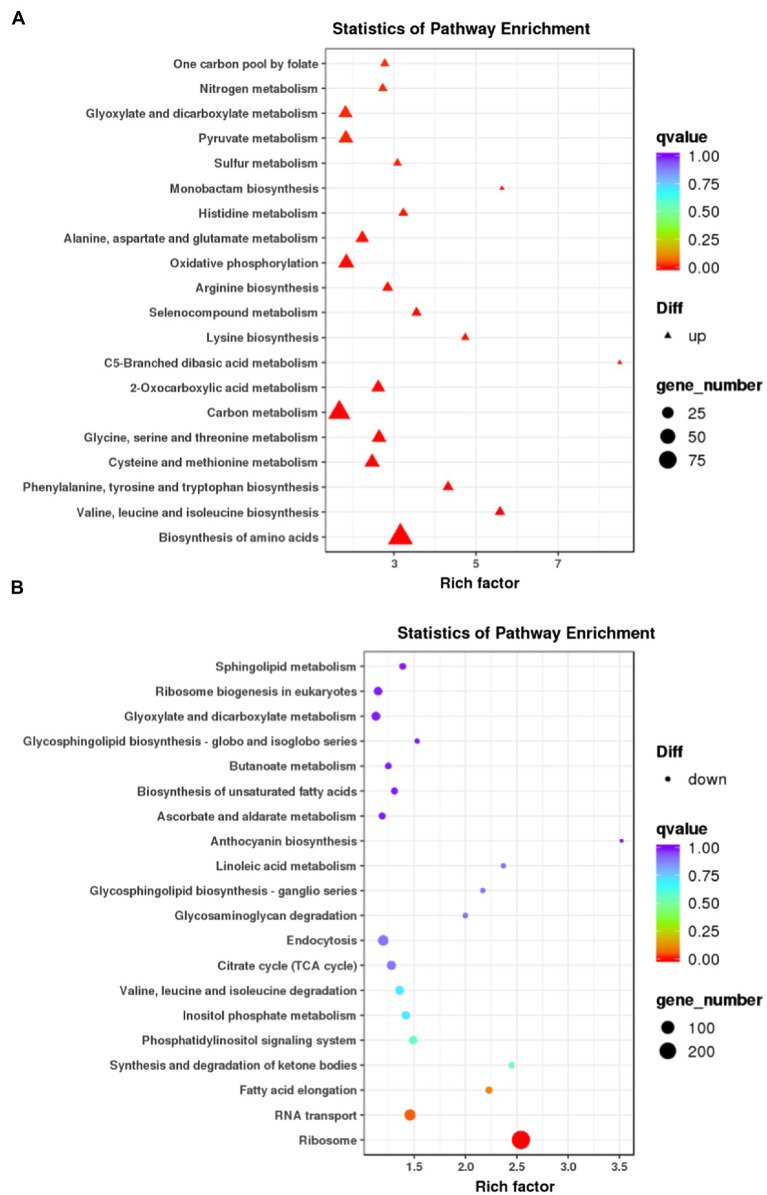
KEGG pathway enrichment analysis of DEGs. **(A)** KEGG of upregulated genes. **(B)** KEGG of downregulated genes. Each circle in the figure represents a KEGG pathway. The *y*-axis represents the pathway name and the *x*-axis represents Enrichment Factor, which represents the ratio of the proportion of genes annotated to a pathway in the differential genes to the proportion of genes annotated to this pathway in all genes.

We next carried out the Gene Ontology (GO) enrichment analysis. In biological process (BP) category, DEGs related to “translation” and “cytoskeleton” were overrepresented (Figure 2A). In both cellular component (CC) and molecular function (MF) categories, the enriched DEGs were mostly related to “ribosome” and “cytoskeleton” (Figures 2B,C). The KEGG pathway analysis showed that most upregulated genes were assigned to “ribosome” and “biosynthesis of amino acids” (Figure 3). It appears that the responses of *R. yunnanense* Franch roots to Al stress occur mainly in intracellular components. However, previous reports of transcriptome analysis on roots of buckwheat and rice showed that genes encoding extracellular-localized proteins were preferentially induced by Al stress (Tsutsui et al., 2012; Zhu et al., 2015; Xu et al., 2017). These results indicated that *R. yunnanense* Franch employed biological processes different from that of crops to cope with Al toxicity.

**Figure 4:**
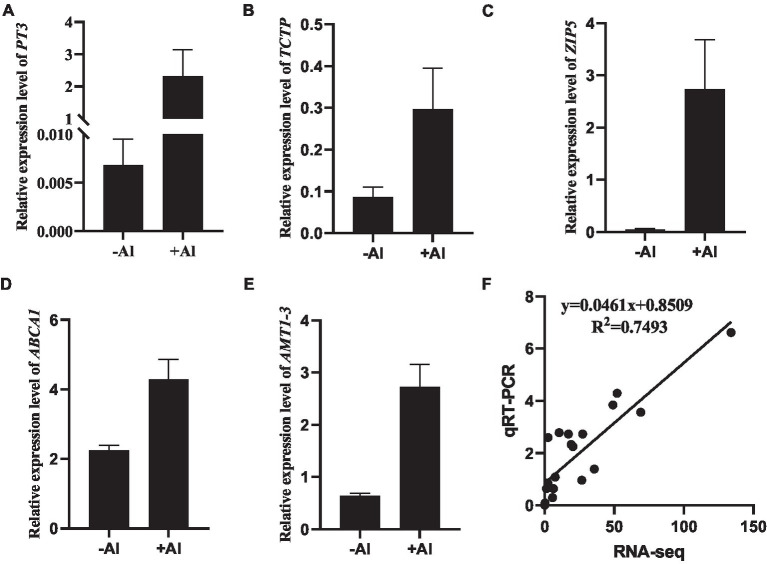
Validation of gene expression by qRT-PCR. **(A–E)** RT-qPCR analysis of gene expression of *PT3* **(A)**, *TCTP* **(B)**, *ZIP5* **(C)**, *ABCA1* **(D)**, and *AMT1-3* **(E)** in root apex of *R. yunnanense* Franch in response to 50 μM Al for 6 h. *ACTIN* was used as an internal control to normalize expression. Data are means ± SD (*n* = 3 for biological repeats). **(F)** Correlation of gene expression levels between RNA-Seq data and qRT-qPCR analysis.

To validate the accuracy of RNA-seq data, a total of 11 genes (Supplementary Table S6) were selected for further quantitative RT-PCR (qRT-PCR) analysis. The expression of five DEGs, namely c281256.graph_c0 (PT3; Figure 4A), c311155.graph_c0 (TCTP; Figure 4B), c273058.graph_c0 (ZIP5; Figure 4C), c293675.graph_c0 (ABCA1; Figure 4D), and c290690.graph_c0 (AMT1-3; Figure 4E), whose expression was upregulated in our RNA-seq data was confirmed *via* qRT-PCR analysis. Overall, the high correlation coefficient (*R*^2^ = 0.7493) between the two analyses (Figure 4F) suggests that the gene expression calculated from RNA-seq data is credible.

### Identification of Al-tolerance gene homologs in *Rhododendron yunnanense* Franch roots

Al-tolerance genes have been identified from model plant species such as *Arabidopsis thaliana* and rice (Yang et al., 2019; Chen et al., 2022). To get insight into the molecular basis of Al-tolerance mechanisms in *R. yunnanense* Franch, we identified genes homologous to known Al-tolerance genes reported previously. As shown in Table 1, there are three genes homologous to *Arabidopsis STOP1* and one gene homologous to *STOP2*; one gene is homologous to *SENSITIVE TO ALUMINUM RHIZOTOXICITY 1* (*STAR1*) and two genes homologous to *STAR2/ALS3*. Moreover, we found three *FDH* homologous genes and seven *AAE3* homologous genes in *R. yunnanense* Franch roots. However, we have not identified genes encoding both citrate-permeable MATE proteins and aluminum-activated malate transporter proteins from *R. yunnanense* Franch roots (Supplementary Tables S4, S5). Additionally, except *STOP1*, most of these Al-tolerance genes are induced by Al stress, and only the expression of *STOP2* and *ALS3;1* was found to be differentially expressed in root apex of *R. yunnanense* Franch under Al stress (Table 1). These results are in agreement with our GO enrichment and KEGG pathway analyses, showing that it might be intracellular biological processes that are implicated in high Al tolerance of *R. yunnanense* Franch. Al-induced organic acid anion efflux, which has been reported to be the most important Al exclusion mechanism, is not implicated in high Al tolerance of *R. yunnanense* Franch.

**Table 1:** Identification of known Al-tolerance gene homologs in RNA-seq.

Gene ID	FPKM value			log2FC	Annotation	Arabidopsis homologous gene (Identity)
	+Al	−Al	FDR			
c302821.graph_c0	35.74	26.63	0.08	0.52	STOP1;1	AT1G34370 (62%)
c309866.graph_c1	41.9	49.32	0.74	−0.14	STOP1;2	AT1G34370 (58%)
c291884.graph_c0	18.1	13.11	0.00	0.56	STOP1;3	AT1G34370 (55%)
c295016.graph_c0	8.24	2.45	0.00	1.65	STOP2	AT5G22890 (50%)
c107438.graph_c1	0.44	0.61	–	–	STAR1	AT1G67940 (58%)
c300169.graph_c0	133.89	69.05	0.00	1.01	ALS3;1	AT2G37330 (87%)
c341743.graph_c0	0.1	0.23	–	–	ALS3;2	AT2G37330 (58%)
c321452.graph_c0	0.61	0.26	–	–	FDH;1	AT5G14780 (76%)
c307333.graph_c1	3.03	2.76	0.89	0.22	FDH;2	AT5G14780 (82%)
c236333.graph_c0	0.46	0.38	0.90	0.31	FDH;3	AT5G14780 (57%)
c304242.graph_c0	7.2	5.92	0.66	0.37	AAE3;1	AT3G48990 (79%)
c593092.graph_c0	0	1.3	–	–	AAE3;2	AT3G48990 (80%)
c301574.graph_c0	174.44	140.35	0.00	0.41	AAE3;3	AT3G48990 (79%)
c453734.graph_c0	0	0.92	–	–	AAE3;4	AT3G48990 (70%)
c143846.graph_c0	0.26	0.15	–	–	AAE3;5	AT3G48990 (63%)
c418906.graph_c0	0	2.01	–	–	AAE3;6	AT3G48990 (61%)
c335291.graph_c0	0.1	0.39	–	–	AAE3;7	AT3G48990 (58%)

### ALS3 is a tonoplast-localized transporter protein

**Figure 5:**
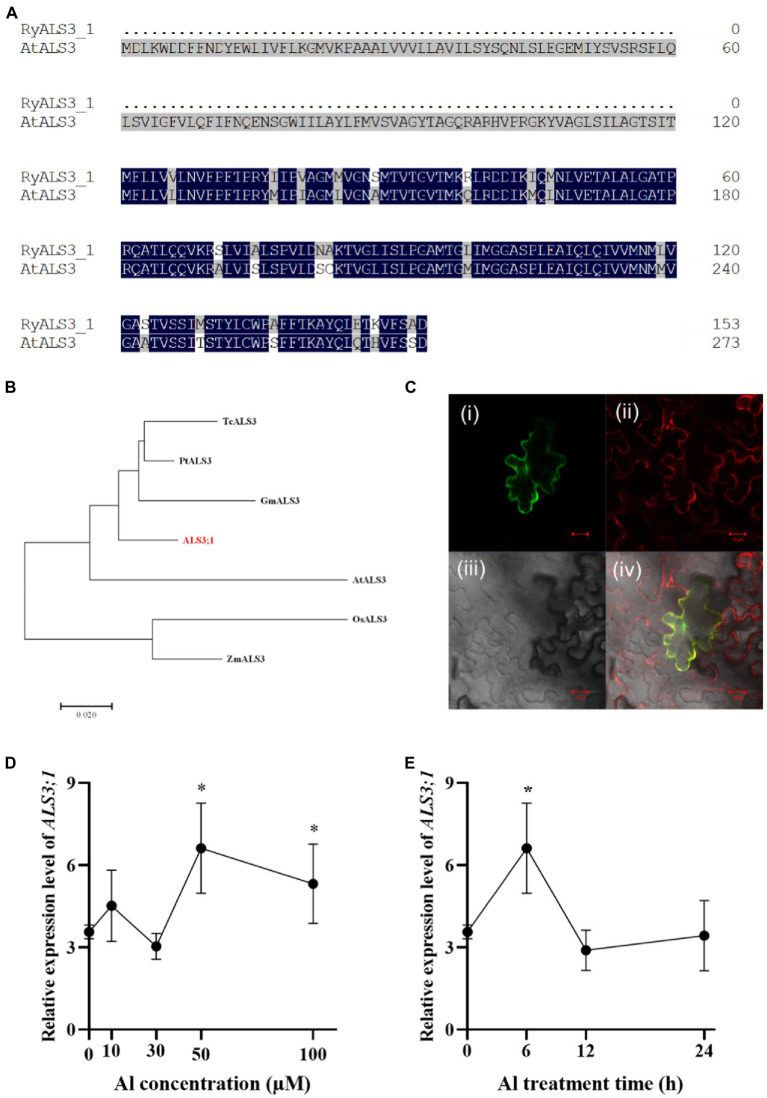
Amino acids alignment, phylogenic analysis, subcellular location, and expression of RyALS3;1 in *R. yunnanense* Franch. **(A)** Amino acid sequence alignment of *R. yunnanense* Franch ALS3;1 protein with Arabidopsis ALS3 (At2g37330). **(B)** Phylogenetic analysis of *R. yunnanense* Franch ALS3;1 and its close homologs. **(C)** RyALS3;1 is localized to the tonoplast. (i) RyALS3;1-GFP (green); (ii) tonoplast marker, vac-rk CD3-975 (red); (iii) bright field; (iv) a merged view of i–iii. Scale bar = 10 μm. **(D–E)** Expression pattern of *R. yunnanense* Franch ALS3;1. Two-month-old seedlings were subjected to 1/5 nutrient Hoagland solution with 0, 10, 30, 50, or 100 μM Al for 6 h **(D)** or with 50 μM Al for different treatment time **(E)**. *ACTIN* was used as an internal control to normalize expression. Data are means ± SD (*n* = 3 for biological repeats). Asterisks (*) represent a significant difference in comparison with 0 μM Al control.

The finding that *R. yunnanense* Franch roots accumulated substantial amounts of Al and the accumulated Al was confined to roots led us to hypothesize that Al immobilization in roots may contribute to this root Al accumulation. In *Arabidopsis*, the ALUMINUM SENSITIVE 3 (ALS3) protein interacts with STAR1 to form a tonoplast-localized ATP-binding cassette (ABC) transporter complex (Dong et al., 2017). Therefore, it is possible that ALS3;1 (c300169.graph_c0) contributes to Al immobilization in roots of *R. yunnanense* Franch. To test this, we first carried out amino acid alignment of RyALS3;1 with AtALS3. *R. yunnanense* ALS3;1 (RyALS3;1) is a protein of 153 amino acids in length, which is much shorter than AtALS3 (Figure 5A). Phylogenic analysis showed that *R. yunnanense Ry*ALS3;1 clustered with those from dicots and separated with monocot ALS proteins (Figure 5B).

Next, we examined the subcellular localization of the RyALS3;1 protein by transiently expressing RyALS3;1-green fluorescence protein (GFP) fusion protein in tobacco (*Nicotiana benthamiana*) leaves (Figure 5C). RyALS3;1-GFP signals were found to be overlapped with the co-expressed tonoplast red fluorescence (Figure 5C) by a laser confocal microscope. Therefore, RyALS3;1 is a tonoplast-localized protein in *R. yunnanense* Franch.

To further validate that RyALS3;1 is implicated in Al tolerance, we performed qRT-PCR analysis of its transcriptional changes by Al stress. In an Al dosage experiment, we found that Al at concentrations as high as 50 μM could induce *RyALS3;1* expression (Figure 5D). In a time-course experiment, the expression of *RyALS3;1* was transiently induced by 50 μM Al (Figure 5E). Therefore, it is possible that *RyALS3;1* represents an early Al-responsive gene contributing to high Al tolerance for *R. yunnanense* Franch.

### ALS3-mediated Al tolerance by compartmentalizing Al into vacuoles

**Figure 6:**
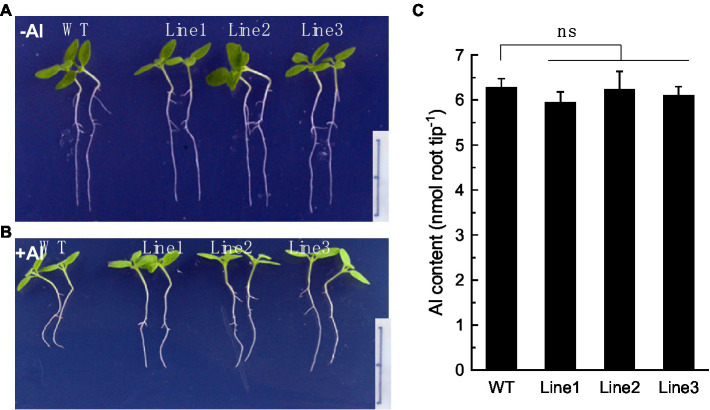
RyALS3;1 confers Al tolerance in transgenic tobacco lines. **(A)** Growth of both WT plants and three independent transgenic plants in the absence of Al. **(B)** Growth of both WT plants and three independent transgenic plants in the presence of 5 μM Al. Note that the transgenic lines have much longer root elongation than WT plants. Bar = 1 cm. **(C)** Al content in root apex of both WT and transgenic lines. Data are means ± SD (*n* = 3). ns: not statistically different.

To confirm that RyALS3;1 is exactly involved in Al root immobilization, we constructed transgenic tobacco plants overexpressing RyALS3;1. Three independent tobacco lines overexpressing RyALS3;1 were used to examine their tolerance to Al stress. Without Al stress, no difference was observed for root elongation between the wild-type (WT) and transgenic lines during 4 days of treatment (Figure 6A). However, the transgenic lines displayed greater primary root elongation than WT plants in the presence of Al (5 μM; Figure 6B), suggesting that expression of RyALS3;1 confers transgenic tobacco higher tolerance to Al stress. To further confirm that RyALS3-mediated Al tolerance is associated with internal tolerance, we measured Al content in the root apex of tobacco plants. However, no significant difference of Al content exists between WT plants and three transgenic lines (Figure 6C), indicating that the improved Al tolerance is related to ALS3-mediated Al immobilization in roots.

## Discussion

The *Rhododendron* is of great ornamental value because of its beauty and diversity of flowers. For example, as many as 40 million pots are required for *R. simsii* hybrids in Belgium annually. Moreover, *Rhododendron* has both medicinal and edible values. For instance, *Rhododendron dauricum* produces daurichromenic acid, a natural product with effective anti-HIV properties (Lee, 2010). Ethnic groups in Yunnan province, China, have regarded eating flowers in spring as a tradition in order to pray for health (Geng et al., 2016). This has greatly promoted the research on *Rhododendron* species (Iijima et al., 2017; Shi et al., 2021). *R. yunnanense* Franch adapts well to acid soils, but the physiological and molecular mechanisms of such adaptation remain unknown. In this present study, we carried out *de novo* transcriptome assembly of *R. yunnanense* Franch. A total of 645,145 transcripts and 443,639 unigenes were identified the from root apex of *R. yunnanense* Franch (Supplementary Table S2). Furthermore, a total of 247,086 were annotated (Supplementary Table S3). Therefore, our results gain a comprehensive overview of *R. yunnanense* Franch transcriptome and provide a basis for further gene identification and functional characterization in terms of Al tolerance.

One of the well-documented Al resistance mechanisms in plants is the Al-activated efflux of organic acid anions from roots (Ryan et al., 2001; Yang et al., 2019). Three kinds of organic acid anions have been frequently reported, i.e., citrate, malate, and oxalate, and genes responsible for citrate and malate efflux have been cloned from a variety of plant species (Chen et al., 2022). Numerous studies have reported that woody plants secrete organic acid anions in response to Al stress. For instance, in cutting roots of *Populus tremula*, both oxalate and citrate efflux were found to be triggered by Al exposure (Qin et al., 2007). Similar findings have also been reported for two coniferous trees, *Cryptomeria japonica* and *Pinus thunbergii* (Hirano et al., 2012). The efflux of malate, citrate, and oxalate in response to Al stress has been observed from the roots of *Populus tremuloides* and *Populus trichocarpa* (Naik et al., 2009). Furthermore, a MATE protein showing 60% identity to AtMATE in amino acid sequence has been identified from *Populus tremula* (Grisel et al., 2010). However, no gene homologous to either citrate-permeable *MATE* gene or malate-permeable *ALMT* gene was identified from our RNA-seq data, suggesting that Al-activated organic acid anions efflux is not implicated in high Al resistance in *R. yunnanense* Franch. However, it remains possible that other organic acids, such as oxalate and succinate, or other substances may be released from roots of *R. yunnanense* Franch to chelate Al. For example, *Camellia sinensis* roots exuded oxalate and caffeine under Al stress. The secretion of phenolic compounds under Al stress has also been observed in *Eucalyptus camaldulensis* and *Melaleuca* species (Nguyen et al., 2003). Therefore, it is necessary to further confirm whether these substances are implicated in high Al resistance in *R. yunnanense* Franch in the future.

We found that Al immobilization in roots is involved in high Al resistance in *R. yunnanense* Franch (Figure 1). To cope with metal toxicity, plants have evolved a diverse sophisticated mechanism. In general, non-accumulating plants employ exclusion strategy to immobilize metals in the root through root cell wall binding or vacuolar compartmentalization. For example, Wang et al. demonstrated that a tonoplast-localized aquaporin protein AtTIP2;2 contributes to immobilizing Zn in the root vacuoles, thereby preventing Zn translocation from root to shoot (Wang et al., 2022). By contrast, accumulating plants cope with excess metals by facilitating translocation of metals from root to shoot, and subsequently sequestering metals in leaf vacuole. In terms of Al accumulation property, woody plants can be classified into Al excluders and Al accumulators (Osaki et al., 1997). Here, we demonstrated that *R. yunnanense* Franch belongs to Al excluders or represents an Al non-accumulator (Figure 1), which is in line with the finding that *R. yunnanense* Franch exhibited high Al tolerance through Al immobilization in roots. Osaki et al. reported that *Melastoma malabathricum* and *Hydrangea macrophylla* could accumulate a large amount of Al in leaves, but *Vaccinium macrocarpon* could accumulate a high amount of Al in roots (Naik et al., 2009). Phylogenetic analyses indicated that Al accumulators predominate in non-flowering plants (Jansen et al., 2002). *R. yunnanense* Franch, as a flowering woody species, is not able to accumulate Al, providing further support to the standpoint of Jansen et al. (2002).

We further revealed the molecular basis of Al immobilization in roots of *R. yunnanense* Franch. We identified RyALS3;1 that was localized to tonoplast and showed sequence similarity to known ALS3 proteins (Figure 5). Moreover, transgenic tobacco plants overexpressing *RyALS3;1* displayed improved Al resistance, but Al content in roots had no difference between WT plants and transgenic plants (Figure 6), implying that the enhanced Al resistance could be attributed to compartmentation of Al within vacuoles. In *Populus tremula*, an *ALS3-like* gene homologous to Arabidopsis *ALS3* gene was identified (Grisel et al., 2010). However, the function of this *ALS3-like* gene in *Populus tremula* has never been characterized. Therefore, we for the first time provided circumstantial evidence that RyALS3;1 is implicated in Al immobilization in roots of *R. yunnanense* Franch. ALS3 was first identified by screening Arabidopsis mutants hypersensitive to Al stress, and seemed to be related to the distribution of Al in root tissues (Larsen et al., 2005). Dong et al. demonstrated that AtSTAR1 and ALS3 form a complex protein localized to tonoplast (Dong et al., 2017). This finding led Godon et al. to speculate that AtSTAR1/ALS3 complex might be an Al transporter (Godon et al., 2019). However, OsSTAR1 and OsSTAR2 (ALS3 homolog) form a complex protein localized to vesicles and possibly transport UDP-glucose from cytosol to cell wall in rice (Huang et al., 2009). Similarly, buckwheat FeSTAR1 and FeALS3 have been reported to form a complex, localized to vesicles, and involved in cell wall modification (Xu et al., 2018, 2019), but two half-size ABC transporters, FeASL1.1 and FeALS1.2, are involved in the internal detoxification of Al by sequestering Al into the vacuoles (Lei et al., 2017). Therefore, whether RyALS3;1 interacts with a STAR1 homolog has to be investigated in the future.

Given that there is a great amount of Al accumulated in vacuoles of root cells of *R. yunnanense* Franch, it is reasonable to deduce that Al must first be transported across the plasma membrane with the aid of plasma membrane-localized transporters having transport activity toward Al. In rice, Nrat1 is a plasma membrane-localized NRMAP family member, which shows substrate specificity to Al (Xia et al., 2010). However, phylogenetic analysis and motif survey revealed that this type of Al transporter exists only within cereal crops (Chen et al., 2022). In this study, we did not identify genes homologous to Nrat1 in our transcriptome of *R. yunnanense* Franch (Table 1). A different type of Al transporter has been identified in Arabidopsis, where the aquaporin protein AtNIP1;2 is responsible for bidirectional transport of Al-malate complex (Wang et al., 2017). However, this type of Al transporter seems not to exist in *R. yunnanense* Franch, which is in agreement with the lacking of ALMT protein in the transcriptome data. Whether there are other transporters responsible for transport of Al across plasma membrane requires further investigation.

In summary, we demonstrated that *R. yunnanense* Franch displays high Al tolerance *via* Al immobilization in roots. Moreover, we found that *R. yunnanense* Franch does not accumulate Al in its leaves. We provided the first whole-genome transcriptome analysis of *R. yunnanense* Franch roots in response to Al stress and identified 443,639 unigenes, which provided a molecular basis for future gene identification and functional characterization. Finally, we revealed the molecular mechanism in which a tonoplast-localized ALS3 protein is responsible for Al sequestration in vacuoles, thus contributing to root Al immobilization in *R. yunnanense* Franch.

## Data availability statement

The original contributions presented in the study are publicly available. This data can be found at: NCBI, PRJNA841036.

## Author contributions

Y-XX and S-HJ conceived the research. Y-XX, Y-SL, S-XH, JZ, and Z-YW performed experiments. Y-XX analyzed bioinformatic data and wrote the manuscript. All authors contributed to the article and approved the submitted version.

## Funding

This work was financially supported by the National Key Research and Development Program of China (2019YFE0118900), the National Natural Science Foundation of China (31971641), the Zhejiang Provincial Natural Science Foundation of China (LY20C160008), the research developmental fund of Jiyang College of Zhejiang Agriculture and Forestry University (RQ2020B15), and the scientific research training program of Jiyang College of Zhejiang Agriculture and Forestry University (202113283005).

## Conflict of interest

The authors declare that the research was conducted in the absence of any commercial or financial relationships that could be construed as a potential conflict of interest.

## Publisher’s note

All claims expressed in this article are solely those of the authors and do not necessarily represent those of their affiliated organizations, or those of the publisher, the editors and the reviewers. Any product that may be evaluated in this article, or claim that may be made by its manufacturer, is not guaranteed or endorsed by the publisher.

## Footnotes

^1^ http://www.genome.jp/kegg/
