# Supplementary material for: Combined de novo transcriptomic and physiological analyses reveal RyALS3-mediated aluminum tolerance in Rhododendron yunnanense Franch
Source: Front Plant Sci. 2022 Aug 10;13:951003. doi: 10.3389/fpls.2022.951003 (PMC9399778; doi:10.3389/fpls.2022.951003)

Figure S1. The correlations of RNA-sequencing biological replicates.

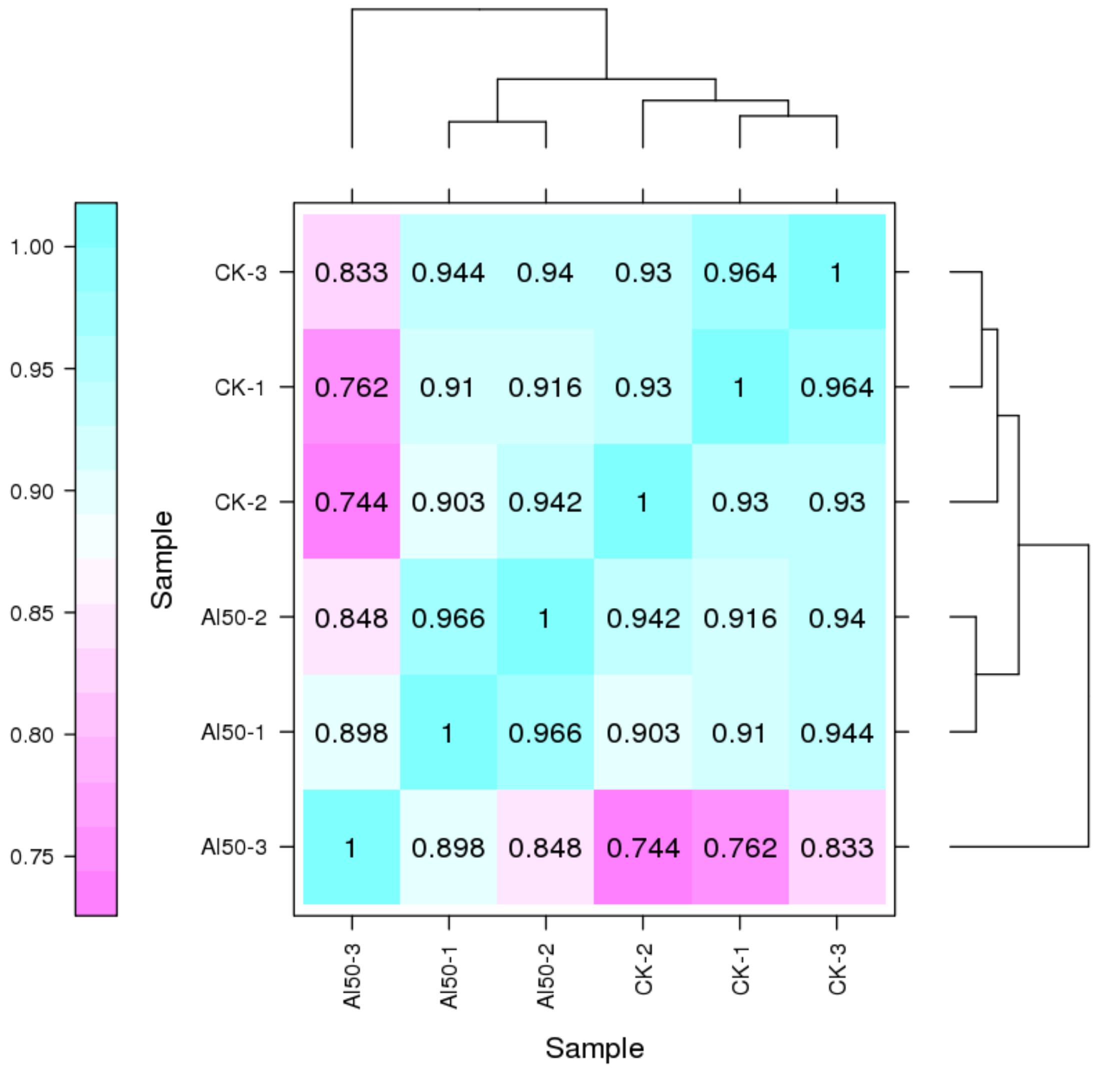

Figure S2. Differentially expressed unigenes (DEGs) in *Rhododendron yunnanense* Franch roots. (A) Volcano map of differentially expressed genes. Each point in the volcano diagram represents a gene, and the horizontal axis represents the log value of multiple difference of a gene expression in the two samples. The greater the absolute value is, the greater the multiple difference of expression between the two samples is. The ordinate represents the negative log value of the error detection rate. The larger the value is, the more significant the differential expression is, and the more reliable the differential expression genes obtained by screening are. (B) Cluster diagram of expression patterns of differentially expressed genes. Different columns

represent different samples, and different rows represent different genes. The color represents the log base 2 of the amount of gene expression FPKM in the sample.

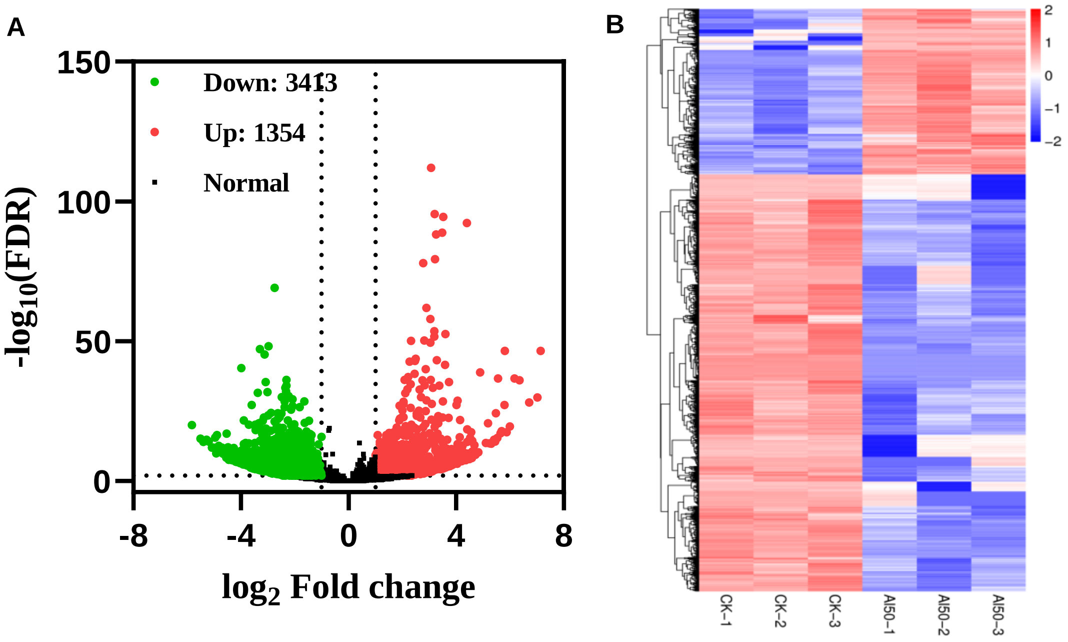

Supplement: Supplementary file 1 [file Data_Sheet_1.PDF]
